# Sociodemographic and behavioural factors of adherence to the no-screen guideline for toddlers among parents from the French nationwide Elfe birth cohort

**DOI:** 10.1186/s12966-022-01342-9

**Published:** 2022-08-12

**Authors:** Lorraine Poncet, Mélèa Saïd, Malamine Gassama, Marie-Noëlle Dufourg, Falk Müller-Riemenschneider, Sandrine Lioret, Patricia Dargent-Molina, Marie-Aline Charles, Jonathan Y. Bernard

**Affiliations:** 1 Centre for Research in Epidemiology and Statistics (CRESS), Université de Paris, Inserm, INRAE, 75004 Paris, France; 2 Unité Mixte Inserm-Ined-EFS Elfe, 93322 Aubervilliers, Ined France; 3 Saw Swee Hock School of Public Health, National University of Singapore, Singapore, Singapore; 4 Berlin Institute of Health, Charite University Medical Centre, Berlin, Germany; 5 Singapore Institute for Clinical Sciences (SICS), Agency for Science, Technology and Research (A*STAR), Singapore, Singapore

**Keywords:** Child, Birth cohort, Parenting, Screen time, Sedentary behavior, Smartphone, Tablet, Television

## Abstract

**Background:**

Excessive screen time in infancy and childhood has been associated with consequences on children’s development and health. International guidelines call for no screen time before age 2 years, whereas in France, the most prominent guidelines recommend no screen before age 3 years. However, data are lacking on parental adherence to the no-screen guideline for toddlers and factors of adherence in France. Using data from the French nationwide Elfe birth cohort, we estimated adherence to the no-screen guideline at age 2 years and examined related factors, including sociodemographic characteristics, parental leisure activities and screen time.

**Methods:**

In 2011, 18,329 newborns and their parents were enrolled in 349 randomly selected maternity units across mainland France. At age 2 years, screen exposure of 13,117 toddlers was reported by parents in phone interviews. Data on sociodemographic characteristics, parental leisure activities and screen time were collected from both parents. Three patterns of parental leisure activities were derived by principal component analysis: literate (e.g.,reading), screen-based, and physical/artistic activities. Multivariable logistic regression models were used to examine the associations of sociodemographic characteristics, parental leisure activities and parental screen time with adherence to the no-screen guideline for toddlers.

**Results:**

Overall, 1809/13,117 (13.5%) families adhered to the no-screen guideline for toddlers. Adherence was reduced with maternal age < 40 years, low parental education, single-parent household and parental migration status. After adjusting for sociodemographic characteristics, adherence to the guideline was positively associated with a parental literate activity pattern (mothers: odds ratio [95% confidence interval]: 1.15 [1.08, 1.22]); fathers: 1.15 [1.07, 1.23]) and negatively with a screen-based activity pattern (mothers: 0.73 [0.69, 0.77]; fathers: 0.81 [0.76, 0.87]). With each additional hour of parental screen time, mothers and fathers were less likely to adhere to the guideline (mothers: adjusted odds ratio 0.80 [0.77, 0.83]; fathers: 0.88 [0.85, 0.91]).

**Conclusions:**

Adherence to the no-screen guideline for toddlers in France was low. Parental leisure activities and parental screen time are major factors of adherence to the no-screen guideline and could be considered in targeted public health interventions.

**Supplementary Information:**

The online version contains supplementary material available at 10.1186/s12966-022-01342-9.

## Background

Screens have gained an increasingly central place in our everyday lives: new types of screen devices have appeared and opportunities to use them have multiplied. In the process, they have also become more present in children’s lives. The adverse effects of screen use in children and toddlers have been widely documented in observational studies. They include increased risk of overweight and obesity [1, 2], reduced sleep duration [3, 4] and impaired language and cognitive development [5–7]. Studies have also stressed the importance of media content, showing better language development with exposure to educational and well-designed TV programs [8, 9], as well as the importance of parental co-viewing and discussing content with parents [10].

Academic societies and national public health agencies have developed guidelines aimed at limiting the time children spend watching screens. In particular, the American Academy of Pediatrics, pioneer in the field, and more recently the World Health Organization recommend that parents do not engage their children < 2 years old in screen activities, and if they do so, the American Academy of Pediatrics recommends limiting exposure to high-quality programs co-viewed with parents [11, 12]. In France, although more recent screen guidelines recommend that children > 2 years old have only limited exposure to programs that should be co-viewed with parents [13–16], the most popular guideline has asserted for more than a decade that children < 3 years old should not engage in any screen viewing [17].

Studies have shown that children’s screen viewing is associated with sociodemographic characteristics [18, 19], including young maternal age [20, 21], low maternal and paternal educational attainment [20, 22–24], and non-white ethnicity [22, 25]. Mixed results were found regarding an association with household income [22, 26]. Existing studies have focused on the role of parents in their child’s screen viewing, showing that parental screen use is positively associated with child screen use [27, 28]. Other aspects studied include parental screen-related practices [29], knowledge of recommendations [28, 30], parental rule-setting on child screen use [28, 31], self-efficacy for limiting child screen use [30, 32], and household media equipment [28].

We considered that parents’ socioeconomic position likely influenced child screen use via several pathways. The first pathway is by knowledge of recommendations, remembering that different social groups receive health promotion messages differently. Health messages and recommendations tend to address concerns (e.g., long-term health) more widely shared by individuals with higher socioeconomic position, who are often in a better position to implement them [33]. Second, insofar as socioeconomic position shapes preferences, tastes and practices, it also shapes leisure time activities [34] and determines the frequency of screen viewing, book reading and other leisure activities within a household. The type of activities parents engage in at home will in turn make it more or less feasible to implement child screen use recommendations. In this study, we aim to investigate the role of parents’ socioeconomic position on child screen use. Unlike previous studies, we focused on the activities that parents engage in during their leisure time (i.e., whether it involves literate, screen viewing, or physical or artistic activities). Since among all parents’ leisure activities, parental screen time is expected to be central in determining adherence to the no screen guideline, we further investigate parental screen time and its association with guideline adherence.

Factors of child screen exposure at age 2 years have not been investigated in France, and few international studies have benefitted from a large, representative sample. Using data from the French nationwide Étude longitudinale française depuis l’enfance (Elfe; French Longitudinal Study of Children) birth cohort, we aimed to 1) estimate adherence to the no-screen guideline for children among parents of toddlers aged 2 years, 2) identify parents’ sociodemographic characteristics and leisure activities associated with guideline adherence, and 3) investigate the association between parental screen time and guideline adherence and its variation according to sociodemographic characteristics.

## Methods

### Study design and population

We used data from the Elfe cohort, a prospective nationally representative birth cohort study initiated in 2011. The general objective of the Elfe cohort was to examine the determinants of the child’s development, health and socialization from birth to adulthood. The study design and protocol have been described in detail [35]. In brief, 18,329 newborns were included from a random sample of 349 maternity units. They were born after 33 weeks’ gestation to mothers > 18 years old who were not planning to move outside of Metropolitan France in the following 3 years and were able to read French, Arabic, Turkish, or English. Participation rate at inclusion was 51%. To correct for non-representativeness, basic information on non-participating mothers was collected and a weighting procedure was developed to account for the sampling plan and non-participation at both maternity ward and individual levels. Mothers provided written consent for their own and their child's participation. Fathers provided written consent for the child's participation when present at inclusion or were informed about their rights to oppose it. The Elfe study was approved by the Advisory Committee for Treatment of Health Research Information (*Comité Consultatif sur le Traitement des Informations pour la Recherche en Santé*), the National Data Protection Authority (CNIL) and the National Statistics Council.

After a face-to-face questionnaire administered to mothers by midwives at inclusion, follow-up surveys were conducted by phone or mail. Here, we used data collected at 2 years by phone interviews with children’s mothers and fathers, when available. The survey at 2 years included responses for 13,528 toddlers. The present analysis was based on a sample of 13,117 toddlers with valid data on screen exposure at age 2 years.

### Adherence to the no-screen guideline for toddlers

The questionnaire assessed the use of four screen devices separately: television, computer or tablet, smartphone, and video game console. Parents were asked how often their child used each screen device and answered on a scale of “every day or most days”, “once or twice a week”, “once or twice a month”, “never or almost never”. Our outcome variable “adherence to the no-screen guideline” was computed by combining the answers to these four questions: when parents indicated “never or almost never” for all devices, we considered that parents adhered to the guideline and the outcome variable was coded as 1. Otherwise, the outcome variable was coded as 0. Because we considered that at age 2 years, the child’s activities are driven by parents (or caretakers), here we refer to the parents’ rather than the child’s adherence to the guideline.

### Factors

#### Household sociodemographic characteristics

We examined maternal age (≤ 30, 31–40, > 40 years), maternal and paternal educational attainment (less than high school, high school graduate to 2 years university, ≥ 3 years university), type of household (parents living together, not living together), parental migration status (both born in France, one parent born abroad, both born abroad), household income (in quintiles), parental employment status (both employed, only father employed, only mother employed, both inactive), area of residence (urban, suburban, rural), birth order (first born, later born), number of children in the household (0, 1–2, > 2), child sex (boy, girl) and season of survey (spring, summer, autumn, winter).

#### Parental leisure activities

Both parents were asked about their frequency (never, 1–2 times per month, 1–2 times per week, every day) of 10 types of leisure activities: watching TV, using a PC/smartphone for leisure, playing video games, practicing sport/exercise, hiking/walking, reading books, reading newspapers, visiting museums, going to a library, and practicing artistic activities. Principal component analysis was used to reduce information while accounting for co-dependency between leisure activities. Separately for mothers and fathers, we obtained three leisure activity patterns (Supplementary table S1) that we labelled “literate activities”, “screen-based activities”, and “physical/artistic activities”. Maternal and paternal patterns were used as explanatory variables of adherence to the no-screen guideline in further analyses.

#### Parental screen time

Both parents were asked how much time they spent watching TV programs and using a computer/tablet/smartphone for leisure on a typical weekday and a typical weekend day. Times on weekdays and weekend days were weighted to obtain an average daily screen time ([weekday × 5 + weekend day × 2]/7). Times for TV and other screens were summed to compute a total screen time.

### Statistical analyses

We present household sociodemographic characteristics with unweighted and weighted percentages. We used simple and multivariable logistic regression models to determine the associations of household sociodemographic characteristics with adherence to the no-screen guideline. We checked collinearity between factors using the variance inflation factor (VIF) and Spearman correlations. Because of collinearity between household income and educational attainment (Spearman correlation coefficient = 0.52, VIF 1.4 to 1.8), household income was not included in the multivariable models. Likewise, because birth order and number of children living in the household covaried (Spearman correlation coefficient = 0.81, VIF 2.6 to 2.7), birth order was not included in the multivariable models. We investigated the associations between parental leisure activities and adherence to the no-screen guideline by using bivariable and multivariable logistic regression models, estimating odds ratios (ORs) and 95% confidence intervals (CIs). We computed the likelihood of adherence to the no-screen guideline for each additional hour of parental screen time. We conducted interaction tests and stratified the analyses on three key sociodemographic characteristics, consistently identified as factors of screen viewing in children in previous studies: maternal age, educational attainment, and parental migration status.

Missing data for factors included in the multivariable models were imputed by multiple imputation and the fully conditional specification method. Five datasets were generated, and the estimates and their 95% CIs were pooled. Both complete-case and imputed analyses were presented for multivariable models. We did not adjust for multiple comparisons [36]. Analyses involved using SAS v9.4 (SAS Institute Inc, Cary, NC, USA).

## Results

A total of 13,117 parent–child dyads provided valid data for adherence to the no-screen guideline. Table 1 describes the main characteristics of the study participants. In brief, 55.2% of mothers were 31 to 40 years old. Nearly one third of mothers (31.6%) and fathers (28.4%) had completed 3 years or more of university; 29.2% of mothers and 33.6% of fathers had not completed high school. Overall, 36.1% of children had no siblings, and 6.9% had more than 2 siblings. A total of 1809/13,117 (13.5%) parents adhered to the no-screen guideline for toddlers. Maternal and paternal total mean screen times were 161 and 157 min/day, respectively.

**Table 1:** Sociodemographic characteristics of families at 2-year postnatal in the Elfe birth cohort

		N	%	Weighted %
Overall sample		13,117	100,0	100,0
Maternal age				
	≤ 30 years	4,216	32.2	38.7
	31–40 years	8,112	61.9	55.2
	> 40 years	774	5.9	6.1
	Missing	15	0.1	
Mother educational attainment				
	Below high school	2,265	17.3	29.2
	Completed high school-2 years university	5,275	40.2	39.2
	≥ 3 years university	5,312	40.5	31.6
	Missing	265	2.0	
Father educational attainment				
	Below high school	3,008	22.9	33.6
	Up to 2 years university	4,600	35.1	37.9
	≥ 3 years university	4,175	31.8	28.4
	Missing	1,334	10.2	
Type of household				
	Parents living together	12,529	95.5	92.0
	Parents not living together	571	4.4	8.0
	Missing	17	0.1	
Parental migration status				
	No immigrant parent	10,850	82.7	74.1
	1 immigrant parent	1,585	12.1	15.2
	2 immigrant parents	665	5.1	10.7
	Missing	17	0.1	
Household income				
	First quintile	2,414	18.4	31.3
	Second quintile	2,530	19.3	21.5
	Third quintile	2,441	18.6	17.3
	Fourth quintile	2,473	18.9	15.9
	Fifth quintile	2,523	19.2	14.0
	Missing	736	5.6	
Parental employment status				
	Both employed	9,895	75.4	70.1
	Only father employed	1,854	14.1	21.4
	Only mother employed	494	3.8	4.5
	Both inactive	253	1.9	4.0
	Missing	621	4.7	
Area of residence				
	Urban	8,141	62.1	64.0
	Suburban	4,480	34.2	32.4
	Isolated	418	3.2	3.6
	Missing	78	0.6	
Birth order				
	First born	5,940	45.3	42.7
	Later born	7,177	54.7	57.4
Child sex				
	Boy	6,659	50.8	50.5
	Girl	6,458	49.2	49.5
Season of survey				
	Spring	1,998	15.2	21.9
	Summer	3,350	25.5	26.4
	Autumn	3,762	28.7	26.5
	Winter	4,007	30.5	25.3
Number of siblings				
	0	4,988	38.0	36.1
	1–2	7,415	56.5	57.0
	> 2	713	5.4	6.9
	Missing	1	0.0	
Type of childcare				
	Parents	3,759	28.7	38.3
	Grand parents	581	4.4	4.3
	Childminder/nanny	5,894	44.9	37.4
	Day-care centre	2,882	22.0	20.0
	Missing	1	0.0	
Adherence to the no-screen guideline		1,809	13.8	13.5
Mean maternal screen time (min/day)		12,874	153	161 (158–165)
Mean paternal screen time (min/day)		10,653	155	157 (154–160)

Three parental leisure activity patterns were identified, accounting for about 40% of the total variance for both mothers and fathers (Supplemental Table 1). The first pattern was positively correlated with book and newspaper reading as well as museum and library attendance for both mothers and fathers; we labelled this pattern “literate activities”. The second pattern, labelled “screen-based activities”, had high positive factor loadings for PC/smartphone use, TV use and videogame use for both mothers and fathers and additionally, newspaper reading for fathers. The third pattern, labelled “physical/artistic activities”, was positively correlated with hiking/walking, sport practice and artistic activities for both mothers and fathers.

In multivariable analyses with multiple imputation, as compared with mothers aged > 40 years old, those aged ≤ 30 years old and 31–40 years were less likely to adhere to the guideline (OR [95% CI]: 0.66 [0.54, 0.82]) and (0.75 [0.62, 0.92], respectively) (Table 2). Likelihood of adhering to the guideline was negatively associated with maternal educational attainment below high school (0.71 [0.59, 0.85]) and up to 2 years of university (0.71 [0.63, 0.81]) versus higher maternal education. A similar association was found for paternal educational attainment (below high school: 0.84 [0.71, 0.99]; up to 2 years of university: 0.74 [0.65, 0.85]). Likelihood of adherence was also negatively associated with both parents being immigrants (0.56 [0.42, 0.74]) versus both being born in France.

**Table 2:** Unadjusted and adjusted associations of sociodemographic characteristics with adherence to the no-screen guideline for toddlers among parents from the Elfe birth cohort

			Unadjusted models with complete cases	Adjusted model with complete cases^a^ (*n* = 11,438)	Adjusted model with multiple imputation^a^ (*n* = 13,117)
		% (n)	OR (95% CI)	aOR (95% CI)	aOR (95% CI)
Maternal age					
	≤ 30 years	11.5 (486)	0.59 (0.48, 0.73)	0.61 (0.48, 0.77)	0.66 (0.54, 0.82)
	31–40 years	14.6 (1,183)	0.77 (0.64, 0.94)	0.70 (0.57, 0.87)	0.75 (0.62, 0.92)
	> 40 years	18.1 (140)	1.00	1.00	1.00
Maternal educational attainment					
	< high school	11.2 (254)	0.61 (0.53, 0.71)	0.70 (0.57, 0.85)	0.71 (0.59, 0.85)
	Completed high school-2 years university	11.5 (606)	0.63 (0.56, 0.70)	0.71 (0.62, 0.80)	0.71 (0.63, 0.81)
	≥ 3 years university	17.1 (909)	1.00	1.00	1.00
Paternal educational attainment					
	Below high school	11.9 (357)	0.65 (0.56, 0.74)	0.81 (0.69, 0.95)	0.84 (0.71, 0.99)
	Completed high school-2 years university	11.4 (526)	0.62 (0.55, 0.70)	0.72 (0.63, 0.82)	0.74 (0.65, 0.85)
	≥ 3 years university	17.3 (720)	1.00	1.00	1.00
Type of household					
	Parents living together	14.1 (1,765)	1.00	1.00	1.00
	Parents not living together	7.5 (43)	0.50 (0.36, 0.68)	0.64 (0.33, 1.23)	0.58 (0.42, 0.80)
Parental migration status					
	No immigrant parent	14.2 (1,542)	1.00	1.00	1.00
	1 immigrant parent	13.1 (208)	0.91 (0.78, 1.07)	0.87 (0.73, 1.04)	0.89 (0.76, 1.05)
	2 immigrant parents	8.7 (58)	0.58 (0.44, 0.76)	0.50 (0.35, 0.71)	0.56 (0.42, 0.74)
Household income					
	First quintile	12.1 (291)	0.80 (0.68, 0.95)		
	Second quintile	13.2 (335)	0.89 (0.76, 1.05)		
	Third quintile	13.6 (333)	0.93 (0.79, 1.09)		
	Fourth quintile	14.6 (362)	1.00 (0.86, 1.18)		
	Fifth quintile	14.6 (368)	1.00		
Parental employment status					
	Both employed	14.2 (1,402)	1.00	1.00	1.00
	Only father employed	13.1 (243)	0.91 (0.79, 1.06)	1.03 (0.85, 1.23)	1.01 (0.85, 1.21)
	Only mother employed	16.0 (79)	1.15 (0.90, 1.48)	1.09 (0.82, 1.45)	1.23 (0.96, 1.58)
	Both inactive	15.4 (39)	1.10 (0.78, 1.56)	1.48 (0.98, 2.25)	1.39 (0.97, 1.99)
Area of residence					
	Urban	13.7 (1,118)	1.02 (0.91, 1.13)	1.40 (1.05, 1.87)	1.31 (1.00, 1.73)
	Suburban	13.5 (607)	1.00	1.00	1.00
	Isolated	16.3 (68)	1.24 (0.94, 1.63)	1.01 (0.90, 1.14)	0.94 (0.84, 1.05)
Birth order					
	First born	13.6 (810)	1.00		
	Later born	13.9 (999)	1.02 (0.93, 1.13)		
Child sex					
	Boy	13.5 (896)	0.94 (0.86, 1.04)	0.92 (0.82, 1.02)	0.93 (0.85, 1.03)
	Girl	14.1 (913)	1.00	1.00	1.00
Number of children living in the household					
	0	15.2 (656)	1.00	1.00	1.00
	1–2	14.1 (1,043)	1.08 (0.97, 1.20)	1.00 (0.89, 1.13)	1.04 (0.93, 1.16)
	> 2	15.4 (110)	1.21 (0.97, 1.50)	1.20 (0.93, 1.55)	1.22 (0.96, 1.54)
Type of childcare					
	Parents	13.0 (487)	1.00	1.00	1.00
	Grandparents	8.6 (50)	0.63 (0.47, 0.86)	0.63 (0.44, 0.90)	0.64 (0.47, 0.88)
	Childminder/nanny	13.8 (814)	1.08 (0.95, 1.22)	0.99 (0.85, 1.16)	0.96 (0.83, 1.11)
	Day-care centre	15.9 (458)	1.27 (1.11, 1.46)	1.13 (0.95, 1.35)	1.13 (0.96, 1.32)
Season of survey					
	Spring	12.4 (248)	0.76 (0.65, 0.89)	0.78 (0.66, 0.93)	0.75 (0.64, 0.89)
	Summer	15.7 (527)	1.00	1.00	1.00
	Autumn	13.4 (504)	0.83 (0.73, 0.95)	0.84 (0.73, 0.97)	0.82 (0.72, 0.94)
	Winter	13.2 (530)	0.82 (0.72, 0.93)	0.80 (0.69, 0.92)	0.81 (0.71, 0.93)

^a^Adjusted models were mutually adjusted for all variables shown in the table except household income and birth order

After adjusting for sociodemographic characteristics in analyses with multiple imputation, adherence to the guideline was positively associated with maternal and paternal literate activity patterns (1.15 [1.08, 1.22] and 1.15 [1.07, 1.23], respectively), and negatively with screen-based activity patterns (0.73 [0.69, 0.77] and 0.81 [0.76, 0.87], respectively) (Table 3). We found no associations between physical/artistic activity patterns and adherence to the guideline.

**Table 3:** Unadjusted and adjusted associations of leisure activity patterns with adherence to the no-screen guideline for toddlers among parents from the Elfe birth cohort

	Unadjusted model with complete cases	Adjusted model with complete case^a^ (*n* = 10,045)	Adjusted model with multiple imputation^a^ (*n* = 13,117)
Patterns	OR (95% CI)	aOR (95% CI)	aOR (95% CI)
Mother’s literate activities	1.30 (1.24, 1.37)	1.23 (1.14, 1.33)	1.15 (1.08, 1.22)
Father’s literate activities	1.59 (1.49, 1.69)	1.23 (1.14, 1.32)	1.15 (1.07, 1.23)
Mother’s screen-based activities	0.68 (0.64, 0.71)	0.69 (0.65, 0.74)	0.73 (0.69, 0.77)
Father’s screen-based activities	0.68 (0.64, 0.72)	0.73 (0.68, 0.78)	0.81 (0.76, 0.87)
Mother’s physical/artistic activities	0.99 (0.94, 1.04)	1.04 (0.97, 1.11)	1.01 (0.95, 1.06)
Father’s physical/artistic activities	1.01 (0.95, 1.07)	1.00 (0.94, 1.07)	0.98 (0.93, 1.04)

^a^aOR were mutually adjusted for all six types of parental practices, and further adjusted for maternal age, maternal and paternal educational attainment, type of household, parental migration status, area of residence, child sex, number of siblings, type of childcare and season or survey

For each additional hour spent by parents using screens daily, adherence to the no-screen guideline for their child was less likely for mothers (0.80 [0.77. 0.83] and fathers (0.88 [0.85. 0.91]) in multiply imputed analyses (Table 4). The decrease in likelihood was sharpest in households with high maternal educational attainment (mothers: 0.72 [0.67. 0.76]; fathers: 0.80 [0.75. 0.84]) and no immigrant parents (mothers: 0.78 [0.75. 0.82]; fathers: 0.87 [0.83. 0.90]).

**Table 4:** Adjusted associations of maternal and paternal screen time (h/day) and adherence to the no-screen guideline, stratified on three key socio-demographic variables, Elfe birth cohort

		Mother’s screen time (h/day)^a^		Father’s screen time (h/day)^a^		Mother’s screen time (h/day)^b^		Father’s screen time (h/day)^b^	
		aOR	Interaction P	aOR	Interaction P	aOR	Interaction P	aOR	Interaction P
Overall		0.78 (0.74, 0.81)		0.80 (0.76, 0.84)		0.80 (0.77, 0.83)		0.88 (0.85, 0.91)	
Maternal age^c^			0.009		0.02		0.01		0.42
	≤ 30 years	0.84 (0.78, 0.90)		0.77 (0.69, 0.85)		0.85 (0.80, 0.90)		0.90 (0.84, 0.96)	
	31–40 years	0.74 (0.70, 0.78)		0.79 (0.75, 0.84)		0.77 (0.73, 0.81)		0.86 (0.82, 0.90)	
	> 40 years	0.78 (0.66, 0.91)		0.96 (0.83, 1.11)		0.84 (0.73, 0.97)		0.96 (0.83, 1.10)	
Maternal educational attainment^d^			< 0.0001		0.003		< 0.0001		< 0.0001
	Below high school	0.89 (0.82, 0.96)		0.84 (0.72, 0.98)		0.90 (0.84, 0.97)		0.94 (0.87, 1.02)	
	Completed high school—2 years university	0.80 (0.74, 0.85)		0.88 (0.82, 0.95)		0.82 (0.78, 0.87)		0.94 (0.88, 0.99)	
	≥ 3 years university	0.70 (0.65, 0.75)		0.74 (0.69, 0.79)		0.72 (0.67, 0.76)		0.80 (0.75, 0.84)	
Parental migration status^e^			0.04		0.08		0.01		0.15
	No immigrant parent	0.76 (0.72, 0.79)		0.79 (0.75, 0.83)		0.78 (0.75, 0.82)		0.87 (0.83, 0.90)	
	1 immigrant parent	0.88 (0.79, 0.98)		0.89 (0.78, 1.03)		0.87 (0.79, 0.96)		0.94 (0.84, 1.06)	
	2 immigrant parents	0.82 (0.64, 1.05)		1.03 (0.75, 1.42)		0.92 (0.78, 1.08)		0.93 (0.74, 1.17)	

^a^Analyses on complete cases

^b^Analyses on multiple imputations

^c^aOR adjusted for maternal and paternal educational attainment, type of household, parental migration status, area of residence, child sex, number of siblings, type of childcare and season of survey

^d^aOR adjusted for maternal age, paternal educational attainment, type of household, parental migration status, area of residence, child sex, number of siblings, type of childcare and season of survey

^e^aOR adjusted for maternal age, maternal and paternal educational attainment, type of household, area of residence, child sex, number of siblings, type of childcare and season of surve

## Discussion

In this nationally representative sample of parents of 2-year olds, we found that the rate of adherence to the no-screen guideline for toddlers was 13.5%. Several sociodemographic characteristics were associated with low adherence to the guideline, including young maternal age, low parental educational attainment, parents not living together, and immigrant parents. Parents were more likely to adhere to the guideline when their own leisure time involved literate activities and were less likely to adhere when their leisure time involved screen-based activities. Parental daily screen time was associated with low likelihood to adhere to the guideline, with differences according to maternal age, maternal educational attainment and migration status.

The rate of adherence to the no-screen guideline implies that 87% of toddlers aged 2 years have some screen viewing, although with varied frequency and duration. International studies estimate screen use in different ways. In a nationally representative US survey, the rate of children < 2 years old who watched screens on a typical day was 68% in 2003 [37] and 90% in 2007 [23]. In Singapore, Goh et al. reported that 88% of children aged 18 to 24 months watched screens daily [28]. For comparison purposes, we computed a similar rate of daily screen use in our sample: 63% of the children used screens daily (at least one of the devices).

Our findings are consistent with previous studies identifying young maternal age [20] and low parental educational attainment [23, 26, 38] as correlates of greater screen time in young children and found no or weak associations with household income [5, 39, 40] and parental employment [5, 26]. Concerning ethnicity and migration background, studies conducted in the United States have reported that screen time was greater for toddlers from Black and Hispanic households compared to toddlers from White households [23, 41]. However, Thompson et al. showed that in Hispanic households, toddlers of English-speaking but not Spanish-speaking Hispanic mothers had higher screen time, which suggests that longer duration of residence or being born in the United States may play a role in child screen exposure [42]. Unlike previous studies [22, 23, 39], we found that guideline adherence was less likely in single-parent households and that a greater number of children in the household was associated with greater guideline adherence. Parental screen time, for both mothers and fathers, was negatively associated with guideline adherence, in line with previous studies [28, 43].

Analysing the role of parental leisure activities allowed for a finer understanding of the link between sociodemographic characteristics and guideline adherence. Provided that parents have knowledge of the guideline to limit screen exposure in young children, its execution is likely to be favoured in family environments that promote leisure activities other than screen-based activities. Specifically, parental literate activities, including reading newspapers and books and attendance at libraries and museums, was related to greater guideline adherence, independent of parental screen-based activities. However, our study suggests that young children’s screen viewing behaviour is independent of their parents’ leisure activities linked to exercise and arts. To our knowledge, this finding is novel and warrants further exploration.

In our study, high parental screen time was associated with low adherence to the no-screen guideline. The association was greatest in households with high maternal educational attainment and no immigrant parent. Thus, in households with low socioeconomic status, adherence to the guideline may be low regardless of parental screen use, whereas in households with high socioeconomic status, toddlers’ exposure to screens may occur when parental screen use increases.

The main strength of our study is that we analysed data from a nationally representative birth cohort with a very large sample size. Original and extensive measurements of leisure activities of both mothers and fathers allowed us to explore these understudied characteristics. We were able to investigate a broad range of sociodemographic variables related to screen exposure in young children, and explore associations of parental screen time and guideline adherence in relation to key sociodemographic variables.

Our study has limitations. Because of social desirability, parents may have under-reported their child’s exposure to screens. Authors have argued that children’s screen time as estimated by parents is moderately correlated with actual screen time [29, 44]. In our study, parents were asked to report the frequency of their child’s screen use, which, because it demands less precision, might be less prone to under-reporting. No psychometric properties were computed for child and parental screen use measurement tools, therefore validity and reliability of the measurements can not be assessed. However, comprehensive recall period (week day and weekend day) and multiple types of screen use assessed enables us to gauge child and parental screen use in its fast-changing variety. Screen exposure of children cared for by a childminder during the day may be underestimated by parents, contrary to children cared for in day-care centres, where screens are generally prohibited. We had no data to assess parental knowledge of the no-screen guideline, which would have been of importance to disentangle awareness of the guideline from the ability to implement the guideline. Last, our data were collected in 2013, when the Elfe children reached 2 years of age. The fiercely publicised national public debate proposes that children’s screen time has increased since 2013, so data collected in the Elfe cohort may already be outdated. Yet, although French national surveys have reported an increase in screen time among French youth aged 11–17 years from the mid-2000s to the mid-2010s, screen time remained relatively stable among French children aged 3–10 years [45–48]. Although on average daily screen time in children aged 3 to 17 years increased by 20 min between 2006 and 2015, the increase concerned mostly boys aged > 10 years and girls aged > 14 years [45]. The present study is the first to report national data on children < 3 years old. In the absence of more recent national data, any speculation on the evolution since 2013 needs to balance both the spread of screens in society and the increased knowledge of screen time guidelines in the general population, given that guidelines have been broadcast at the national level. More recent surveys are needed to evaluate knowledge of screen time guidelines. Screen use guideline for toddlers, like all public health guidelines, are likely to be received and implemented differently across social groups: more privileged groups are likely to have greater awareness of the recommendations and implement them more readily. Therefore, it is possible that social inequalities in screen use at 2 years may have increased in the last 10 years. In this case, the associations we found between social characteristics and screen use at 2 years could be underestimated.

## Conclusions

In this nationally representative sample, 13.5% of parents adhered to the no-screen guideline for toddlers. We identified sociodemographic factors, parental leisure activities and screen time as factors of guideline adherence. This knowledge may be important on a national scale to promote parental adherence to the no-screen guideline and reframe screen use as an issue that concerns the whole family, not just the child. Intervention studies may benefit from targeting parental screen time and promoting alternative activities at the household level, promoting parental strategies, including modelling, for reducing opportunities for sedentary behaviours, thus increasing opportunities for physical activity.

## Supplementary Information

**Additional file 1.**

**Additional file 2.**

## Data Availability

The datasets generated and analysed during the current study are not publicly available for reasons of privacy for the participants. SAS scripts are available from the corresponding author upon reasonable request. Established researchers who would like access to the data from the Elfe cohort study can request them to the Committee of Access to the Data from the Elfe cohort on the website of the survey: www.elfe-france.fr.
