# Supplementary material for: Sociodemographic and behavioural factors of adherence to the no-screen guideline for toddlers among parents from the French nationwide Elfe birth cohort
Source: Int J Behav Nutr Phys Act. 2022 Aug 12;19:104. doi: 10.1186/s12966-022-01342-9 (PMC9373389; doi:10.1186/s12966-022-01342-9)
Supplement: Supplementary file 1 — Additional file 1. [file 12966_2022_1342_MOESM1_ESM.docx]

**Sociodemographic and behavioural correlates of adherence to the no-screen guideline for toddlers among parents from the French nationwide Elfe birth cohort**

**Table 1**. Sociodemographic characteristics of families at 2-year postnatal in the Elfe birth cohort

|  |  | N | % | Weighted % |
| --- | --- | --- | --- | --- |
| Overall sample | | 13,117 | 100,0 | 100,0 |
| Maternal age | |  |  |  |
|  | ≤30 years | 4,216 | 32.2 | 38.7 |
|  | 31-40 years | 8,112 | 61.9 | 55.2 |
|  | >40 years | 774 | 5.9 | 6.1 |
|  | Missing | 15 | 0.1 |  |
| Mother educational attainment | |  |  |  |
|  | Below high school | 2,265 | 17.3 | 29.2 |
|  | Completed high school-2 years university | 5,275 | 40.2 | 39.2 |
|  | ≥3 years university | 5,312 | 40.5 | 31.6 |
|  | Missing | 265 | 2.0 |  |
| Father educational attainment | |  |  |  |
|  | Below high school | 3,008 | 22.9 | 33.6 |
|  | Up to 2 years university | 4,600 | 35.1 | 37.9 |
|  | ≥3 years university | 4,175 | 31.8 | 28.4 |
|  | Missing | 1,334 | 10.2 |  |
| Type of household | |  |  |  |
|  | Parents living together | 12,529 | 95.5 | 92.0 |
|  | Parents not living together | 571 | 4.4 | 8.0 |
|  | Missing | 17 | 0.1 |  |
| Parental migration status | |  |  |  |
|  | No immigrant parent | 10,850 | 82.7 | 74.1 |
|  | 1 immigrant parent | 1,585 | 12.1 | 15.2 |
|  | 2 immigrant parents | 665 | 5.1 | 10.7 |
|  | Missing | 17 | 0.1 |  |
| Household income | |  |  |  |
|  | First quintile | 2,414 | 18.4 | 31.3 |
|  | Second quintile | 2,530 | 19.3 | 21.5 |
|  | Third quintile | 2,441 | 18.6 | 17.3 |
|  | Fourth quintile | 2,473 | 18.9 | 15.9 |
|  | Fifth quintile | 2,523 | 19.2 | 14.0 |
|  | Missing | 736 | 5.6 |  |
| Parental employment status | |  |  |  |
|  | Both employed | 9,895 | 75.4 | 70.1 |
|  | Only father employed | 1,854 | 14.1 | 21.4 |
|  | Only mother employed | 494 | 3.8 | 4.5 |
|  | Both inactive | 253 | 1.9 | 4.0 |
|  | Missing | 621 | 4.7 |  |
| Area of residence | |  |  |  |
|  | Urban | 8,141 | 62.1 | 64.0 |
|  | Suburban | 4,480 | 34.2 | 32.4 |
|  | Isolated | 418 | 3.2 | 3.6 |
|  | Missing | 78 | 0.6 |  |
| Birth order | |  |  |  |
|  | First born | 5,940 | 45.3 | 42.7 |
|  | Later born | 7,177 | 54.7 | 57.4 |
| Child sex | |  |  |  |
|  | Boy | 6,659 | 50.8 | 50.5 |
|  | Girl | 6,458 | 49.2 | 49.5 |
| Season of survey | |  |  |  |
|  | Spring | 1,998 | 15.2 | 21.9 |
|  | Summer | 3,350 | 25.5 | 26.4 |
|  | Autumn | 3,762 | 28.7 | 26.5 |
|  | Winter | 4,007 | 30.5 | 25.3 |
| Number of siblings | |  |  |  |
|  | 0 | 4,988 | 38.0 | 36.1 |
|  | 1-2 | 7,415 | 56.5 | 57.0 |
|  | >2 | 713 | 5.4 | 6.9 |
|  | Missing | 1 | 0.0 |  |
| Type of childcare | |  |  |  |
|  | Parents | 3,759 | 28.7 | 38.3 |
|  | Grand parents | 581 | 4.4 | 4.3 |
|  | Childminder/nanny | 5,894 | 44.9 | 37.4 |
|  | Day-care centre | 2,882 | 22.0 | 20.0 |
|  | Missing | 1 | 0.0 |  |
|  |  |  |  |  |
| Adherence to the no-screen guideline | | 1,809 | 13.8 | 13.5 |
|  | |  |  |  |
| Mean maternal screen time (min/day) | | 12,874 | 153 | 161 (158-165) |
| Mean paternal screen time (min/day) | | 10,653 | 155 | 157 (154-160) |

**Table 2**. Unadjusted and adjusted associations of sociodemographic characteristics with adherence to the no-screen guideline for toddlers among parents from the Elfe birth cohort

|  |  |  | Unadjusted models with complete cases | Adjusted model with complete cases^1^ (n=11,438) | Adjusted model with multiple imputation^1^  (n=13,117) |
| --- | --- | --- | --- | --- | --- |
|  |  | % (n) | OR (95% CI) | aOR (95% CI) | aOR (95% CI) |
| Maternal age | |  |  |  |  |
|  | ≤30 years | 11.5 (486) | 0.59 (0.48, 0.73) | 0.61 (0.48, 0.77) | 0.66 (0.54, 0.82) |
|  | 31-40 years | 14.6 (1,183) | 0.77 (0.64, 0.94) | 0.70 (0.57, 0.87) | 0.75 (0.62, 0.92) |
|  | >40 years | 18.1 (140) | 1.00 | 1.00 | 1.00 |
| Maternal educational attainment | |  |  |  |  |
|  | <high school | 11.2 (254) | 0.61 (0.53, 0.71) | 0.70 (0.57, 0.85) | 0.71 (0.59, 0.85) |
|  | Completed high school-2 years university | 11.5 (606) | 0.63 (0.56, 0.70) | 0.71 (0.62, 0.80) | 0.71 (0.63, 0.81) |
|  | ≥3 years university | 17.1 (909) | 1.00 | 1.00 | 1.00 |
| Paternal educational attainment | |  |  |  |  |
|  | Below high school | 11.9 (357) | 0.65 (0.56, 0.74) | 0.81 (0.69, 0.95) | 0.84 (0.71, 0.99) |
|  | Completed high school-2 years university | 11.4 (526) | 0.62 (0.55, 0.70) | 0.72 (0.63, 0.82) | 0.74 (0.65, 0.85) |
|  | ≥3 years university | 17.3 (720) | 1.00 | 1.00 | 1.00 |
| Type of household | |  |  |  |  |
|  | Parents living together | 14.1 (1,765) | 1.00 | 1.00 | 1.00 |
|  | Parents not living together | 7.5 (43) | 0.50 (0.36, 0.68) | 0.64 (0.33, 1.23) | 0.58 (0.42, 0.80) |
| Parental migration status | |  |  |  |  |
|  | No immigrant parent | 14.2 (1,542) | 1.00 | 1.00 | 1.00 |
|  | 1 immigrant parent | 13.1 (208) | 0.91 (0.78, 1.07) | 0.87 (0.73, 1.04) | 0.89 (0.76, 1.05) |
|  | 2 immigrant parents | 8.7 (58) | 0.58 (0.44, 0.76) | 0.50 (0.35, 0.71) | 0.56 (0.42, 0.74) |
| Household income | |  |  |  |  |
|  | First quintile | 12.1 (291) | 0.80 (0.68, 0.95) |  |  |
|  | Second quintile | 13.2 (335) | 0.89 (0.76, 1.05) |  |  |
|  | Third quintile | 13.6 (333) | 0.93 (0.79, 1.09) |  |  |
|  | Fourth quintile | 14.6 (362) | 1.00 (0.86, 1.18) |  |  |
|  | Fifth quintile | 14.6 (368) | 1.00 |  |  |
| Parental employment status | |  |  |  |  |
|  | Both employed | 14.2 (1,402) | 1.00 | 1.00 | 1.00 |
|  | Only father employed | 13.1 (243) | 0.91 (0.79, 1.06) | 1.03 (0.85, 1.23) | 1.01 (0.85, 1.21) |
|  | Only mother employed | 16.0 (79) | 1.15 (0.90, 1.48) | 1.09 (0.82, 1.45) | 1.23 (0.96, 1.58) |
|  | Both inactive | 15.4 (39) | 1.10 (0.78, 1.56) | 1.48 (0.98, 2.25) | 1.39 (0.97, 1.99) |
| Area of residence | |  |  |  |  |
|  | Urban | 13.7 (1,118) | 1.02 (0.91, 1.13) | 1.40 (1.05, 1.87) | 1.31 (1.00, 1.73) |
|  | Suburban | 13.5 (607) | 1.00 | 1.00 | 1.00 |
|  | Isolated | 16.3 (68) | 1.24 (0.94, 1.63) | 1.01 (0.90, 1.14) | 0.94 (0.84, 1.05) |
| Birth order | |  |  |  |  |
|  | First born | 13.6 (810) | 1.00 |  |  |
|  | Later born | 13.9 (999) | 1.02 (0.93, 1.13) |  |  |
| Child sex | |  |  |  |  |
|  | Boy | 13.5 (896) | 0.94 (0.86, 1.04) | 0.92 (0.82, 1.02) | 0.93 (0.85, 1.03) |
|  | Girl | 14.1 (913) | 1.00 | 1.00 | 1.00 |
| Number of children living in the household | |  |  |  |  |
|  | 0 | 15.2 (656) | 1.00 | 1.00 | 1.00 |
|  | 1-2 | 14.1 (1,043) | 1.08 (0.97, 1.20) | 1.00 (0.89, 1.13) | 1.04 (0.93, 1.16) |
|  | >2 | 15.4 (110) | 1.21 (0.97, 1.50) | 1.20 (0.93, 1.55) | 1.22 (0.96, 1.54) |
| Type of childcare | |  |  |  |  |
|  | Parents | 13.0 (487) | 1.00 | 1.00 | 1.00 |
|  | Grandparents | 8.6 (50) | 0.63 (0.47, 0.86) | 0.63 (0.44, 0.90) | 0.64 (0.47, 0.88) |
|  | Childminder/nanny | 13.8 (814) | 1.08 (0.95, 1.22) | 0.99 (0.85, 1.16) | 0.96 (0.83, 1.11) |
|  | Day-care centre | 15.9 (458) | 1.27 (1.11, 1.46) | 1.13 (0.95, 1.35) | 1.13 (0.96, 1.32) |
| Season of survey | |  |  |  |  |
|  | Spring | 12.4 (248) | 0.76 (0.65, 0.89) | 0.78 (0.66, 0.93) | 0.75 (0.64, 0.89) |
|  | Summer | 15.7 (527) | 1.00 | 1.00 | 1.00 |
|  | Autumn | 13.4 (504) | 0.83 (0.73, 0.95) | 0.84 (0.73, 0.97) | 0.82 (0.72, 0.94) |
|  | Winter | 13.2 (530) | 0.82 (0.72, 0.93) | 0.80 (0.69, 0.92) | 0.81 (0.71, 0.93) |

^1^ Adjusted models were mutually adjusted for all variables shown in the table except household income and birth order.

**Table 3**. Unadjusted and adjusted associations of leisure activity patterns with adherence to the no-screen guideline for toddlers among parents from the Elfe birth cohort

|  | Unadjusted model with complete cases | Adjusted model with complete case^1^ (n=10,045) | Adjusted model with multiple imputation^1^ (n=13,117) |
| --- | --- | --- | --- |
| Patterns | OR (95% CI) | aOR (95% CI) | aOR (95% CI) |
| Mother’s literate activities | 1.30 (1.24, 1.37) | 1.23 (1.14, 1.33) | 1.15 (1.08, 1.22) |
| Father’s literate activities | 1.59 (1.49, 1.69) | 1.23 (1.14, 1.32) | 1.15 (1.07, 1.23) |
| Mother’s screen-based activities | 0.68 (0.64, 0.71) | 0.69 (0.65, 0.74) | 0.73 (0.69, 0.77) |
| Father’s screen-based activities | 0.68 (0.64, 0.72) | 0.73 (0.68, 0.78) | 0.81 (0.76, 0.87) |
| Mother’s physical/artistic activities | 0.99 (0.94, 1.04) | 1.04 (0.97, 1.11) | 1.01 (0.95, 1.06) |
| Father’s physical/artistic activities | 1.01 (0.95, 1.07) | 1.00 (0.94, 1.07) | 0.98 (0.93, 1.04) |

^1^aOR were mutually adjusted for all six types of parental practices, and further adjusted for maternal age, maternal and paternal educational attainment, type of household, parental migration status, area of residence, child sex, number of siblings, type of childcare and season or survey

**Table 4.** Adjusted associations of maternal and paternal screen time (h/day) and adherence to the no-screen guideline, stratified on three key socio-demographic variables, Elfe birth cohort

|  |  | Mother’s screen time (h/day)^1^ | | Father’s screen time (h/day)^1^ | | Mother’s screen time (h/day)^2^ | | Father’s screen time (h/day)^2^ | |
| --- | --- | --- | --- | --- | --- | --- | --- | --- | --- |
|  |  | aOR | Interaction tests | aOR | Interaction tests | aOR | Interaction tests | aOR | Interaction tests |
| Overall | | 0.78 (0.74, 0.81) |  | 0.80 (0.76, 0.84) |  | 0.80 (0.77, 0.83) |  | 0.88 (0.85, 0.91) |  |
| Maternal age^3^ | |  | 0.009 |  | 0.02 |  | 0.01 |  | 0.42 |
|  | ≤30 years | 0.84 (0.78, 0.90) |  | 0.77 (0.69, 0.85) |  | 0.85 (0.80, 0.90) |  | 0.90 (0.84, 0.96) |  |
|  | 31-40 years | 0.74 (0.70, 0.78) |  | 0.79 (0.75, 0.84) |  | 0.77 (0.73, 0.81) |  | 0.86 (0.82, 0.90) |  |
|  | >40 years | 0.78 (0.66, 0.91) |  | 0.96 (0.83, 1.11) |  | 0.84 (0.73, 0.97) |  | 0.96 (0.83, 1.10) |  |
| Maternal educational attainment^4^ | |  | <0.0001 |  | 0.003 |  | <0.0001 |  | <0.0001 |
|  | Below high school | 0.89 (0.82, 0.96) |  | 0.84 (0.72, 0.98) |  | 0.90 (0.84, 0.97) |  | 0.94 (0.87, 1.02) |  |
|  | Completed high school - 2 years university | 0.80 (0.74, 0.85) |  | 0.88 (0.82, 0.95) |  | 0.82 (0.78, 0.87) |  | 0.94 (0.88, 0.99) |  |
|  | ≥3 years university | 0.70 (0.65, 0.75) |  | 0.74 (0.69, 0.79) |  | 0.72 (0.67, 0.76) |  | 0.80 (0.75, 0.84) |  |
| Parental migration status^5^ | |  | 0.04 |  | 0.08 |  | 0.01 |  | 0.15 |
|  | No immigrant parent | 0.76 (0.72, 0.79) |  | 0.79 (0.75, 0.83) |  | 0.78 (0.75, 0.82) |  | 0.87 (0.83, 0.90) |  |
|  | 1 immigrant parent | 0.88 (0.79, 0.98) |  | 0.89 (0.78, 1.03) |  | 0.87 (0.79, 0.96) |  | 0.94 (0.84, 1.06) |  |
|  | 2 immigrant parents | 0.82 (0.64, 1.05) |  | 1.03 (0.75, 1.42) |  | 0.92 (0.78, 1.08) |  | 0.93 (0.74, 1.17) |  |

^1^Analyses on complete cases; ^2^Analyses on multiple imputations; ^3^aOR adjusted for maternal and paternal educational attainment, type of household, parental migration status, area of residence, child sex, number of siblings, type of childcare and season of survey; ^4^aOR adjusted for maternal age, paternal educational attainment, type of household, parental migration status, area of residence, child sex, number of siblings, type of childcare and season of survey; ^5^aOR adjusted for maternal age, maternal and paternal educational attainment, type of household, area of residence, child sex, number of siblings, type of childcare and season of survey

**Supplemental Table 1**. Factor loadings from principal component analysis of mothers’ and fathers’ leisure activities in the Elfe birth cohort

|  | Mothers’ leisure activities | | | Fathers’ leisure activities | | |
| --- | --- | --- | --- | --- | --- | --- |
|  | 1^st^ pattern | 2^nd^ pattern | 3^rd^ pattern | 1^st^ pattern | 2^nd^ pattern | 3^rd^ pattern |
| TV use^1^ | -0.17 | **0.62** | 0.12 | -0.21 | **0.59** | 0.23 |
| PC/smartphone use^1^ | 0.20 | **0.67** | -0.05 | 0.21 | **0.69** | -0.02 |
| Video game use^1^ | -0.03 | **0.61** | -0.05 | -0.03 | **0.64** | -0.11 |
| Sport practice^1^ | 0.29 | 0.01 | **0.42** | 0.09 | 0.07 | **0.63** |
| Hiking/walking practice^1^ | 0.01 | 0.00 | **0.74** | 0.02 | -0.05 | **0.78** |
| Book reading^1^ | **0.61** | 0.02 | 0.08 | **0.65** | -0.08 | 0.10 |
| Newspaper reading^1^ | **0.52** | 0.24 | 0.05 | **0.50** | **0.31** | 0.08 |
| Library attendance^2^ | **0.59** | -0.17 | 0.14 | **0.65** | -0.12 | 0.09 |
| Museum attendance^2^ | **0.67** | -0.07 | -0.03 | **0.60** | 0.05 | 0.10 |
| Artistic activities^1,3^ | 0.01 | 0.00 | **0.70** | 0.18 | 0.00 | **0.36** |
|  |  |  |  |  |  |  |
| Explained variance (%) | 17.1 | 13.0 | 11.2 | 17.9 | 13.5 | 10.5 |
|  |  |  |  |  |  |  |
| Pattern label | “Mother’s literate activities” | “Mother’s screen-based activities” | “Mother’s physical and artistic activities” | “Father’s literate activities” | “Father’s screen-based activities” | “Father’s physical and artistic activities” |

^1^Categorised as: Never, 1-2 times per month, 1-2 times per week, or Every day; ^2^In the last 12 months; ^3^Activities defined as drawing, singing, dancing, playing musical instruments, etc.

In bold, factor loadings <-0.30 or >0.30.
